# Real-World Evidence in Cardio-Oncology: What Is It and What Can It Tell Us?∗

**DOI:** 10.1016/j.jaccao.2022.02.002

**Published:** 2022-03-15

**Authors:** Hiroshi Ohtsu, Akihiko Shimomura, Kazuhiro Sase

**Affiliations:** a Leading Center for the Development and Research of Cancer Medicine, Juntendo University, Tokyo, Japan; b Clinical Pharmacology and Regulatory Science, Graduate School of Medicine, Juntendo University, Tokyo, Japan; c Institute for Medical Regulatory Science, Organization for University Research Initiatives, Waseda University, Tokyo, Japan; d Department of Breast and Medical Oncology, National Center for Global Health and Medicine, Tokyo, Japan

**Keywords:** cancer survivorship, cardiovascular disease, real-world evidence, real-world data, risk factors

**Figure:**
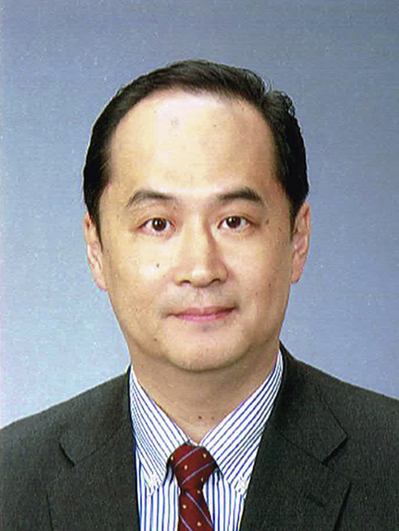

The progress in science depends on techniques, discoveries, and ideas, probably in that order.

—Sydney Brenner (1927-2019)^1^

Galileo created a telescope to study the bright spots—stars—in the night sky. Four centuries later, scientists aimed the Hubble Space Telescope at the dark, deep field and discovered clusters of galaxies.^2^ In the present day, what kind of technology do we need to explore the burgeoning new frontier between cardiology and oncology?

The incidence of cardiovascular disease (CVD) in cancer survivors^3^^,^^4^ and the incidence of cancer in patients with CVD^5^^,^^6^ are increasing along with life expectancy. In addition, CVD and cancer share risk factors—such as smoking, obesity, and diabetes—presumably because of similar underlying mechanisms, such as chronic inflammation.^7^^,^^8^ However, the causal relationship between CVD and 3 key elements—the patient, cancer, and cancer treatment—remains controversial.

First, each patient has his or her specific risk factors for CVD. The battle against the CVD pandemic began in earnest in the mid-20th century, when CVD emerged as the leading cause of death.^9^ Among the most far-reaching early research studies is the Framingham Heart Study, which identified risk factors including smoking, hypertension, and dyslipidemia; it remains the cornerstone of modern research for the prevention, diagnosis, and treatment of CVD.

Second, cancer itself is a multifaceted risk factor for survivors that includes physical, mental, and socioeconomic aspects.^10^ However, the concept of cancer survivorship emerged only in the mid-1980s^11^ and is much newer than the established CVD risk factors. As such, there is an urgent need to increase research involving long-term (>5 years) cancer survivors, including CVD outcomes in patients with cancer.^10^

The third element is cancer treatment–related CVD (CTRCD). In addition to the traditional cardiotoxicity of radiotherapy and anthracycline-based chemotherapy, molecularly targeted drugs and immune checkpoint inhibitors have led to the emergence of a wide variety of novel cardiovascular disorders, including heart failure, ischemic heart disease, hypertension, valvular heart disease, arrhythmia, thromboembolism, peripheral arterial disease, and pericardial disease.^12^

Because clinical trials in oncology often exclude patients with CVD, and most randomized control trials on CVD do not collect detailed cancer-related information, cardio-oncology desperately needs an alternative methodology to meet the growing unmet medical needs.

In this issue of *JACC: CardioOncology*, Paterson et al^13^ present a detailed population-based retrospective cohort study of the incidence of CVD in adult patients with cancer using an administrative medical database of 4,519,243 adults living in Alberta, Canada. Participants newly diagnosed with cancer during the study period were compared with those without cancer to determine the risk for subsequent cardiovascular events (cardiovascular death, myocardial infarction, stroke, heart failure, and pulmonary embolism) using a time-to-event survival model after adjusting for sociodemographic data and comorbidities.

The investigators conclude that a new cancer diagnosis is independently associated with a significantly increased risk for cardiovascular mortality and nonfatal morbidity, regardless of the cancer site, and highlight the need for a collaborative approach to health care tailored to cancer patients and survivors.

The work by Paterson et al^13^ has remarkable strengths, including large administrative databases with validated risk factors and outcome indicators. The investigators hypothesized that cardiovascular risk increases in all cancer types and is not restricted to heart failure, because excess cardiovascular morbidity due to cancer treatment results from direct myocardial and vascular damage and indirect adverse lifestyle effects. The investigators then used multiple health data repositories in Alberta to construct a large population-based cohort with extensive cancer and CVD profiling. In this retrospective observational study, the risk for cardiovascular events among individuals was compared with and without a history of cancer after adjusting for baseline cardiovascular risk and other elements. The results revealed that a new cancer diagnosis was associated with an increased risk for fatal and nonfatal cardiovascular events, even after adjustment for baseline risk. Therefore, the investigators conclude that, regardless of cancer site, patients were at increased risk for cardiovascular mortality, heart failure, stroke, or pulmonary embolism, which could persist for up to 10 years for heart failure and pulmonary embolism. In addition, patients with cancers of the genitourinary, thoracic, hematologic, gastrointestinal, and nervous systems were identified as high-risk groups warranting further study.

Although the clinical implications of the findings are important, some additional steps are needed before these can be translated into clinical practice. First, the investigators acknowledge that data on cancer therapies, patient ethnicity, and some risk factors for atherosclerosis—such as smoking and physical activity—were not available.^13^ Therefore, unmeasured confounders remain in 2 key elements: CVD risk factors and CTRCD. Second, although the investigators tried to minimize bias, the effect of the sharp difference between groups (median age 56 years [IQR: 43-67 years] vs 34 years [IQR: 23-49 years]) (see Table 1 of Paterson et al^13^) remains even after age and sex adjustments, including >10% differences in hypertension, obesity, diabetes, and peripheral arterial disease (see Supplemental Table 6 of Paterson et al^13^). Third, a population-based analysis has limitations from several perspectives, including strength, consistency, specificity, temporality, biological gradient, and validity.^6^^,^^14^ For example, the HRs of cancer for CVD death (1.33; 95% CI: 1.29-1.37)^13^ may be weaker than the HR of smoking for lung cancer (19.11; 95% CI: 15.8-23.18).^6^ The broad definition of exposure (cancer) and outcome (CVD) may have lowered the specificity of the association. Temporality may need further consideration, because the investigators’ conclusion that incident CVD is higher in the first 12 months focused only on the acute and direct signals, overlooking the late and indirect effects. Moreover, the biological gradient may need further consideration, as no dose-response analysis with quantitative measures of cancer chemotherapy, radiation, or smoking was included.

What cardio-oncology lessons can we learn from these population-based data? Hindsight is 20/20, and it is easy to lament missing data while continuing to remain downstream. However, as the second law of thermodynamics suggests, entropy increases. Therefore, perhaps the lesson we need to learn from Paterson et al^13^ is that it is time for cardiology and oncology to collaborate in order to travel upstream and build a powerhouse to generate information from the new flow of data efficiently.

Recently, the U.S. Food and Drug Administration proposed a new methodology: real-world evidence (RWE).^15^ By definition, real-world data (RWD) are the routinely collected data from various sources, while RWE is the clinical evidence derived from RWD analysis. Each data source—including electronic health records, patient registries, administrative claims–based data, and patient-generated data—has advantages and disadvantages.^16^ However, robust research needs triangulation with many lines of evidence.^17^ Therefore, new methodologies are being developed to optimize the detection of actionable signals.

In sum, the analysis by Paterson et al^13^ highlights the relationship between cancer and CVD. The investigators should be congratulated for their pioneering effort to encourage us to extend our scope beyond the bright spots of known CVD risk factors and CTRCD to include dark and unexplored areas as well. They have taken advantage of an existent data source—clinically validated population-based data in Canada—and tried to provide a bird’s-eye view to analyze factors related to the patient, cancer, and cancer treatment. However, although their conclusion is not inconsistent with natural history or biological facts, there are known limitations, including unmeasured confounding factors inherent to the currently available data sources and methodologies. Cardiology and oncology need to collaborate to launch and successfully execute projects to establish new techniques to use RWD for RWE.

## Funding Support and Author Disclosures

This study was supported in part by a Japan Society for the Promotion of Science/Ministry of Education, Culture, Sports, Science and Technology (KAKENHI 18K12134 to Mr Ohtsu and Dr Sase and 20K08427 to Mr Ohtsu and Dr Sase), the Ministry of Health, Labour and Welfare (20FA1018 to Mr Ohtsu and Dr Sase and 20KC2009 for Dr Sase), and the Agency for Medical Research and Development (20ck0106633h0001 to Mr Ohtsu and Dr Sase). Dr. Shimomura has reported that he has no relationships relevant to the contents of this paper to disclose.

## References

[1] Kenyon C. (2019). Sydney Brenner (1927-2019). Science.

[2] Stofan E. (2021). Hubble’s successor, at last. Science.

[3] Armenian S.H., Xu L., Ky B. (2016). Cardiovascular disease among survivors of adult-onset cancer: a community-based retrospective cohort study. J Clin Oncol.

[4] Armenian S.H., Armstrong G.T., Aune G. (2018). Cardiovascular disease in survivors of childhood cancer: insights into epidemiology, pathophysiology, and prevention. J Clin Oncol.

[5] Bertero E., Robusto F., Rulli E. (2022). Cancer incidence and mortality according to pre-existing heart failure in a community-based cohort.. J Am Coll Cardiol CardioOnc..

[6] Klimis H., Mukherjee S.D., Leong D.P. (2022). What cardio-oncology lessons can we learn from population-based data?. J Am Coll Cardiol CardioOnc..

[7] Koene R.J., Prizment A.E., Blaes A., Konety S.H. (2016). Shared risk factors in cardiovascular disease and cancer. Circulation.

[8] Mehta L.S., Watson K.E., Barac A. (2018). Cardiovascular disease and breast cancer: where these entities intersect: a scientific statement from the American Heart Association. Circulation.

[9] Braunwald E. (1997). Shattuck lecture—cardiovascular medicine at the turn of the millennium: triumphs, concerns, and opportunities. N Engl J Med.

[10] Rowland J.H., Gallicchio L., Mollica M., Saiontz N., Falisi A.L., Tesauro G. (2019). Survivorship science at the NIH: lessons learned from grants funded in fiscal year 2016. J Natl Cancer Inst.

[11] Mullan F. (1985). Seasons of survival: reflections of a physician with cancer. N Engl J Med.

[12] Curigliano G., Lenihan D., Fradley M. (2020). Management of cardiac disease in cancer patients throughout oncological treatment: ESMO consensus recommendations. Ann Oncol.

[13] Paterson D.I., Wiebe N., Cheung W.Y. (2022). Incident cardiovascular disease among adults with cancer: a population-based cohort study. J Am Coll Cardiol CardioOnc.

[14] Hill A.B. (2016). The environment and disease: association or causation?. Proc R Soc Med.

[15] Sherman R.E., Anderson S.A., Dal Pan G.J. (2016). Real-world evidence—what is it and what can it tell us?. N Engl J Med.

[16] Nabhan C., Klink A., Prasad V. (2019). Real-world evidence-what does it really mean?. JAMA Oncol.

[17] Munafò M.R., Davey Smith G. (2018). Robust research needs many lines of evidence. Nature.

